# Porcine reproductive and respiratory syndrome virus degrades TANK-binding kinase 1 via chaperon-mediated autophagy to suppress type I interferon production and facilitate viral proliferation

**DOI:** 10.1186/s13567-024-01392-w

**Published:** 2024-11-14

**Authors:** Shuang-shuang Zhao, Qisheng Qian, Yao Wang, Songlin Qiao, Rui Li

**Affiliations:** 1 Key Laboratory of Applied Technology On Green-Eco-Healthy Animal Husbandry of Zhejiang Province, Zhejiang Provincial Engineering Laboratory for Animal Health Inspection & Internet Technology, Zhejiang International Science and Technology Cooperation Base for Veterinary Medicine and Health Management, China-Australia Joint Laboratory for Animal Health Big Data Analytics, College of Animal Science and Technology & College of Veterinary Medicine of Zhejiang A&F University, Hangzhou, 311300 Zhejiang China; 2 Institute for Animal Health (Key Laboratory of Animal Immunology), Henan Academy of Agricultural Sciences, Zhengzhou, 450002 Henan China

**Keywords:** PRRSV, Nsp2, TBK1, IFN-I, CMA

## Abstract

Porcine reproductive and respiratory syndrome virus (PRRSV) has led to significant economic losses in the global swine industry. Type I interferon (IFN-I) plays a crucial role in the host’s resistance to PRRSV infection. Despite extensive research showing that PRRSV employs multiple strategies to antagonise IFN-I induction, the underlying mechanisms remain to be fully elucidated. In this study, we have discovered that PRRSV inhibits the production of IFN-I by degrading TANK-binding kinase 1 (TBK1) through chaperon-mediated autophagy (CMA). From a mechanistic standpoint, PRRSV nonstructural protein 2 (Nsp2) increases the interaction between the heat shock protein member 8 (HSPA8) and TBK1. This interaction leads to the translocation of TBK1 into lysosomes for degradation, mediated by lysosomal-associated membrane protein 2A (LAMP2A). As a result, the downstream activation of IFN regulatory factor 3 (IRF3) and the production of IFN-I are hindered. Together, these results reveal a new mechanism by which PRRSV suppresses host innate immunity and contribute to the development of new antiviral strategies against the virus.

## Introduction

Innate immunity is the host’s first line of defence against RNA viruses [1, 2]. It recognises viral RNAs through pattern recognition receptors and activates IFN regulatory factor 3 (IRF3) or the nuclear factor kappa-light-chain-enhancer of activated B cells (NF-κB)-mediated type I interferon (IFN-I) production [3–6]. The expression of IFN-I is crucial in limiting the early replication and spread of viruses [7]. RNA viruses have developed different mechanisms to suppress innate immunity and establish infection [8].

Porcine reproductive and respiratory syndrome (PRRS) is characterised by severe reproductive failure in sows and respiratory diseases in pigs of all ages. This disease has imposed a significant economic burden on the global swine industry [9, 10]. Its causative agent, PRRS virus (PRRSV), is a member of the family *Arteriviridae* in the order *Nidovirales.* It is an enveloped, single-stranded positive-sense RNA virus containing a genome of ~15 kb [11, 12]. Two-thirds of the PRRSV genome encodes nonstructural proteins (Nsps), including Nsp1α, Nsp1β, and Nsp2-12, which participate in viral pathogenesis [13–15].

Nsps have been shown to antagonise the host’s innate immune responses and facilitate PRRSV persistent infection in vivo (e.g., porcine alveolar macrophages [PAMs]) and in vitro (such as African green monkey kidney cell lines MA-104 and MARC-145) [16–19]. PRRSV Nsp1α, Nsp4, and Nsp11 all block NF-κB nuclear translocation or eliminate the linear ubiquitination of NF-κB essential modulators, thereby impairing signalling activation and IFN-I production [20–24]. Nsp1β and Nsp2 inhibit the phosphorylation and nuclear translocation of IRF3, which hinders IFN-I expression [25, 26]. Despite these, few studies have previously investigated upstream regulators of IRF3, such as TANK-binding kinase 1 (TBK1).

In this study, we discovered that PRRSV infection leads to the degradation of TBK1. We then identified PRRSV Nsp2 as the factor responsible for TBK1 degradation and outlined the detailed mechanisms involved. Additionally, we found that the degradation of TBK1 by Nsp2 suppresses IRF3-mediated IFN-I production, which in turn facilitates the proliferation of PRRSV.

## Materials and methods

### Cells and viruses

MARC-145, human embryonic kidney 293 T (HEK-293 T), CRL-2843-CD163, and human cervix carcinoma HeLa cells were stored in our laboratory [27]. CRL-2843-CD163 cells were maintained in Roswell Park Memorial Institute 1640 medium (Cat. No. 31800; Solarbio), supplemented with 10% heat-inactivated fetal bovine serum (FBS; Cat. No. 10270–106; Gibco) and antibiotics (100 U/mL penicillin, 100 mg/mL streptomycin; Cat. No. P1400; Solarbio) in a humidified 37 °C and 5% CO_2_ incubator. MARC-145 and HEK-293 T cells were maintained in Dulbecco-modified Eagle medium (Cat. No. 12100; Solarbio), supplemented with 10% heat-inactivated FBS and antibiotics. HeLa cells were routinely maintained in modified Eagle medium (Cat. No. 138–0012; iCell Bioscience Inc), supplemented with 10% heat-inactivated FBS and antibiotics at 37 °C in 5% CO_2_.

PRRSV strains HN07-1 (GenBank KX766378.1) and HNhx (GenBank KX766379) were previously isolated by our laboratory [28, 29]. PRRSV strain BJ-4 (GenBank AF331831) was kindly provided by Professor Hanchun Yang of China Agricultural University. PRRSV strain HN07-1 was utilised in this study unless otherwise stated.

### Antibodies

Rabbit anti-TBK1 monoclonal antibody (mAb; Cat. No. 3504S), rabbit anti-phospho-TBK1 (p-TBK1) mAb (Cat. No. 5483S), rabbit anti-IRF3 mAb (Cat. No. 11904S), rabbit anti-phospho-IRF3 (p-IRF3) mAb (Cat. No. 79945S), rabbit anti-Flag mAb (Cat. No. 14793), mouse anti-myc mAb (Cat. No. 2276S), and rabbit anti-hemagglutinin (HA) mAb (Cat. No. 3724) were all purchased from Cell Signaling Technology. Rabbit anti-heat shock protein member 8 (HSPA8) polyclonal antibodies (pAbs; Cat. No. 10654–1-AP), mouse anti-lysosomal-associated membrane protein 2A (LAMP2A) mAb (Cat. No. 66301–1-Ig), rabbit anti-β-actin mAb (Cat. No. 81115–1-RR), and mouse anti-glyceraldehyde-3-phosphate dehydrogenase (GAPDH) mAb (Cat. No. 60004–1-Ig) were purchased from Proteintech. Alexa Fluor 555-goat anti-rabbit IgG pAbs (Cat. No. ab150078), Alexa Fluor 488-goat anti-rabbit IgG pAbs (Cat. No. ab150077), Alexa Fluor 647-goat anti-rabbit IgG pAbs (Cat. No. ab150115), horseradish peroxidase (HRP)-labeled goat anti-rabbit IgG pAbs (Cat. No. ab6721), and HRP-labeled goat anti-mouse IgG pAbs (Cat. No. ab6789) were purchased from Abcam. Rabbit anti-PRRSV nucleocapsid (N) protein pAbs (Cat. No. GTX129270) for immunoblotting (IB) was purchased from GeneTex.

### Reagents

Lipofectamine 2000 (Cat. No. 2066194), Lipofectamine LTX with Plus reagent (Cat. No. 15338030), and Lipofectamine RNAiMAX transfection reagent (Cat. No. 13778150) were purchased from Invitrogen. TransIntro® PL Transfection Reagent (Cat. No. FT301-01) was purchased from TransGen Biotech. DMSO (Cat. No. 276855), 3-methyladenine (3-MA; Cat. No. M9281), and MG132 (Cat. No. M7449) were purchased from Sigma-Aldrich. Radioimmunoprecipitation assay (RIPA) lysis buffer (Cat. No. P0013B), NP-40 lysis buffer (Cat. No. P0013F), Western blot (WB) /immunoprecipitation (IP) lysis buffer (Cat. No. P0013), fast silver stain kit (Cat. No. P0017S), Triton X-100 (Cat. No. P0096), and 4’,6-diamidino-2-phenylindole (DAPI; Cat. No. C1006) were all purchased from Beyotime Biotechnology. 0.25% trypsin–EDTA solution (Cat. No. T1320), phosphate buffered solution (PBS; Cat. No. P1010), and 4% paraformaldehyde (PFA; Cat. No. P1110) were purchased from Solarbio. A complete EDTA-free protease inhibitor cocktail (Cat. No. 04693116001) and universal SYBR green Master (Cat. No. 04913914001) were purchased from Roche. 2 × loading buffer (Cat. No. 9173), PrimeScript RT master mix (Cat. No. RR036B), and RNAiso Plus (Cat. No. 9109) were purchased from TaKaRa. Enhanced chemiluminescence (ECL) reagent (Cat. No. P0013B) was purchased from NCM Biotechnology. Chloroquine (CQ; Cat. No. HY-17589A), HA beads (Cat. No. HY-K0201), myc beads (Cat. No. HY-K0206-5), and Flag beads (Cat. No. HY-K0207-5) were purchased from MedChemExpress. PolyI:C (Cat. No. P9582) and 0.22 µm polyvinylidene fluoride membranes (Cat. No. ISEQ00010) were obtained from Sigma-Aldrich. The cell viability assay was performed according to our previous study (data not shown) [30].

### Plasmid constructs and transfection

The gene of each Nsp or Nsp2-mutant from PRRSV strain HN07-1 was synthesised and cloned into the pCAGGS-HA plasmid. Nsp2 from PRRSV strains BJ-4 and HNhx was cloned into the pCAGGS-HA plasmid. The cDNA of TBK1 was constructed into pCMV-3 × Flag plasmid by GENEWIZ. The full-length HSPA8 was constructed into the pcDNA3.1-myc/his_A and pEGFP-C1 plasmid by our laboratory [31].

MARC-145, HeLa, and HEK-293 T cells were seeded in cell culture plates and grown to 60~70% confluence for the transfection. Following the manufacturers' instructions, the cells were then transfected with the plasmids using Lipofectamine 2000 or TransIntro PL Transfection Reagent. Unless otherwise specified, 12-well cell culture plates were transfected with 1.5 µg of each plasmid per well, and six-well cell culture plates were transfected with 2.5 µg of each plasmid per well.

### Quantitative real-time PCR (RT-qPCR)

Total RNAs were extracted using TRIzol reagent (Cat. No.15596018; Invitrogen), and then reversely transcribed into cDNA using a PrimeScript RT reagent kit (Cat. No. RR037A) in accordance with the manufacturer’s instructions. RT-qPCR amplified the cDNAs from different samples to quantify N abundance using GAPDH mRNA as an endogenous control. The RT-qPCR was performed using a universal SYBR green master on LightCycler480 II (Roche, Basel, Switzerland). The fold change was calculated using the 2^−△△CT^ method [32]. The primers are listed in Table 1.

**Table 1:** **The primers used for RT-qPCR in this study**

Primers	Sequence (5′-3′)
mon-TBK1-F mon-TBK1-R mon-IFN-β-F mon-IFN-β-R mon-GAPDH-F mon-GAPDH-R pig-HSPA8-F pig-HSPA8-R	CGGAGACCCGGCTGGTATAA GCAGTAGCTCCTTGGCCTAAA CTAGCACTGGCTGGAATGAGACT GGCCTTCAGGTAATGCAGAATC TGACAACAGCCTCAAGATCG GTCTTCTGGGTGGCAGTGAT CGCAGACGTTCACCACCTAT GGAGGTATGCCCGTGAGTTC
pig-LAMP2-F pig-LAMP2-R pig-IFN-β-F pig-IFN-β-R pig-GAPDH-F pig-GAPDH-R	GACTGTTTCAGTGTCTGGAGC TCATCCAGCGAACACTCTTGG TGCAACCACCACAATTCC CTGAGAATGCCGAAGATCTG CCTTCCGTGTCCCTACTGCCAAC GACGCCTGCTTCACCACCTTCT
N-F N-R	AAACCAGTCCAGAGGCAAGG GCAAACTAAACTCCACAGTGTAA

### Immunoblotting (IB)

The cells were harvested and then lysed on ice using WB/IP lysis buffer containing a protease inhibitor cocktail. Whole-cell lysates were normalised to equal amounts of GAPDH/β-actin, separated by sodium dodecyl sulfate–polyacrylamide gel electrophoresis (SDS-PAGE), and transferred onto 0.22 µm polyvinylidene fluoride membranes. The membranes were blocked in 5% skimmed milk at room temperature (RT) for 2 h and then incubated with the primary antibodies at 4 °C overnight. After incubation with the corresponding HRP-conjugated secondary antibodies at RT for 1 h, the IB results were visualised using ECL reagent. Representative images were provided.

### Co-immunoprecipitation (co-IP)

The indicated plasmids were expressed in HEK-293 T cells for 36 h. The cells were lysed with WB/IP lysis buffer containing a protease inhibitor cocktail at 4 °C for 30 min and centrifuged at 12 000 × *g* for 10 min. The supernatant was incubated overnight with anti-HA magnetic beads, anti-Flag magnetic beads, or anti-myc magnetic beads at 4 °C. The precipitated immune complexes were collected with a magnetic holder, washed with cold PBS, eluted with a 2 × loading buffer, and subjected to IB with the indicated antibodies.

### Confocal microscopy

MARC-145 and HeLa cells were transfected with the indicated plasmids or infected with PRRSV for indicated periods. The cells were washed with PBS, fixed with 4% PFA at RT for 10 min, and permeabilised with 0.1% Triton X-100 in PBS at RT for 10 min. After three rinses with cold PBS, the cells were blocked with 5% bovine serum albumin at RT for 1.5 h. After blocking, the cells were incubated overnight with the primary antibodies at 4 °C. After three washes with PBS, the cells were incubated with the appropriate fluorescent secondary antibodies at RT for 1 h. The cell nuclei were stained with DAPI for an additional 5 min. All images were taken and processed using a fluorescence microscope (LSM800, Carl Zeiss AG, Oberkochen, Germany) with a confocal laser scanning setup (20 × , 40 × , or 63 ×). The images represent a single slice from a stack obtained from three independent experiments [33]. The co-localisation analyses were conducted using the JaCoP plugin in ImageJ software following established research guidelines [34–36]. Pearson’s correlation coefficient (> 0.5) describes the correlation of the intensity distribution between channels. Manders’ correlation coefficient (> 0.6) indicates the actual overlap of the signals, representing the true degree of colocalisation [36, 37].

### HA-IP/mass spectrometry (MS)

HEK-293 T cells were transfected with the plasmid expressing Nsp2-HA or HA-tagged empty vector using Lipofectamine 2000 for 36 h, and then were lysed on ice with WB/IP lysis buffer for 30 min. The cell lysates were centrifuged at 12 000 × *g* for 5 min. Anti-HA magnetic beads were incubated overnight with the supernatants at 4 °C. The beads were washed six times with PBS. The associated proteins were analysed by 12.5% SDS-PAGE, and the protein bands in the gel were stained with a fast silver stain kit. The protein bands were excised and subjected to liquid chromatography and tandem MS (LC–MS/MS) by Lumingbio (Shanghai, China). The top-ranked peptide matches were considered for protein identification, and representative images were displayed.

### RNA interference

HEK-293 T cells were transfected with small interference RNA (siRNA) targeting HSPA8 (siHSPA8) or LAMP2A (siLAMP2A) using Lipofectamine RNAiMAX according to the manufacturer’s instructions at a final concentration of 50 nM for 36 h. For *HSPA8* knockdown during PRRSV infection, MARC-145 or CRL-2843-CD163 cells were infected with PRRSV at a multiplicity of infection (MOI) of 1 for 2 h, and then transfected with siHSPA8 or siRNA-negative control (siNC) for 48 h. For *LAMP2A* knockdown, MARC-145 or CRL-2843-CD163 cells were transfected with siLAMP2A or siNC. At 24 h post-transfection, the cells were infected with PRRSV at an MOI of 1. RT-qPCR or IB detected the knockdown efficiencies. The siRNAs were designed and synthesised by GenePharma (Shanghai, China). The cells were then used for subsequent experiments. The siRNA sequences are listed in Table 2. The cell viability assay was performed according to our previous study (data not shown) [30].

**Table 2:** **The siRNAs used in this study**

Name	Forward sequence (5′-3′)	Reverse sequence (5’-3’)
siHSPA8(mon)	GGGACAAGGUAUCAUCAAATT	UUUGAUGAUACCUUGUCCCTT
siLAMP2(mon)	GUGGCACUGUGACAUAUAATT	UUAUAUGUCACAGUGCCACTT
siHSPA8(pig) siLAMP2(pig) siNC	GACCCAGACUUUCACUACUTT GGUUACCUCAGUUAUUAAUTT UUCUCCGAACGUGUCACGUTT	AGUAGUGAAAGUCUGGGUCTT AUUAAUAACUGAGGUAACCTT ACGUGACACGUUCGGAGAATT

### Flow cytometry (FCM)

The MARC-145 cells infected with PRRSV were treated with 0.25% trypsin–EDTA solution, centrifuged at 2000 × *g*, and re-suspended in PBS. The cells were fixed with 4% PFA, permeabilised with 0.1% Triton X-100, and then blocked with 5% bovine serum albumin. The cells were incubated with or without mouse anti-N mAb at RT for 2 h, and Alexa Fluor 647-goat anti-mouse IgG pAbs at RT for 1 h. Finally, the washed cells were resuspended in PBS and the percentage of PRRSV-infected cells was analysed by a flow cytometer (CytoFLEX; Beckman Coulter, Brea, USA). Representative images were shown.

### PRRSV titration assay

The PRRSV-infected cells were subjected to three freeze–thaw cycles and centrifuged to collect the supernatant. The progeny virus titers were measured by detecting the 50% tissue culture infected dose (TCID_50_) in MARC-145 cells. In brief, MARC-145 cells were seeded in 96-well cell culture plates and inoculated with a tenfold serial dilution of the supernatant at 37 °C for 1 h. The excess inoculum was removed by washing it with PBS. The cells were cultured for 3 to 6 days. Cytopathic effects were observed using an inverted microscope (Axiovert 40, Carl Zeiss AG, Oberkochen, Germany). The TCID_50_ value was calculated according to the method of Reed-Muench [38].

### Statistical analysis

Each experiment included three replicates and was independently repeated at least three times. The data are presented as means ± standard error of the mean (SEM). Prism 8.0 software (San Diego, USA) performed all data and calculations using the unpaired two-tailed Student *t* test. Statistical significance was represented by asterisks (ns, not significant [*P* > 0.05]; *, *P* < 0.05; **, *P* < 0.01; ***, *P* < 0.001; ****, *P* < 0.0001).

## Results

### PRRSV Nsp2 degrades TBK1

**Figure 1:**
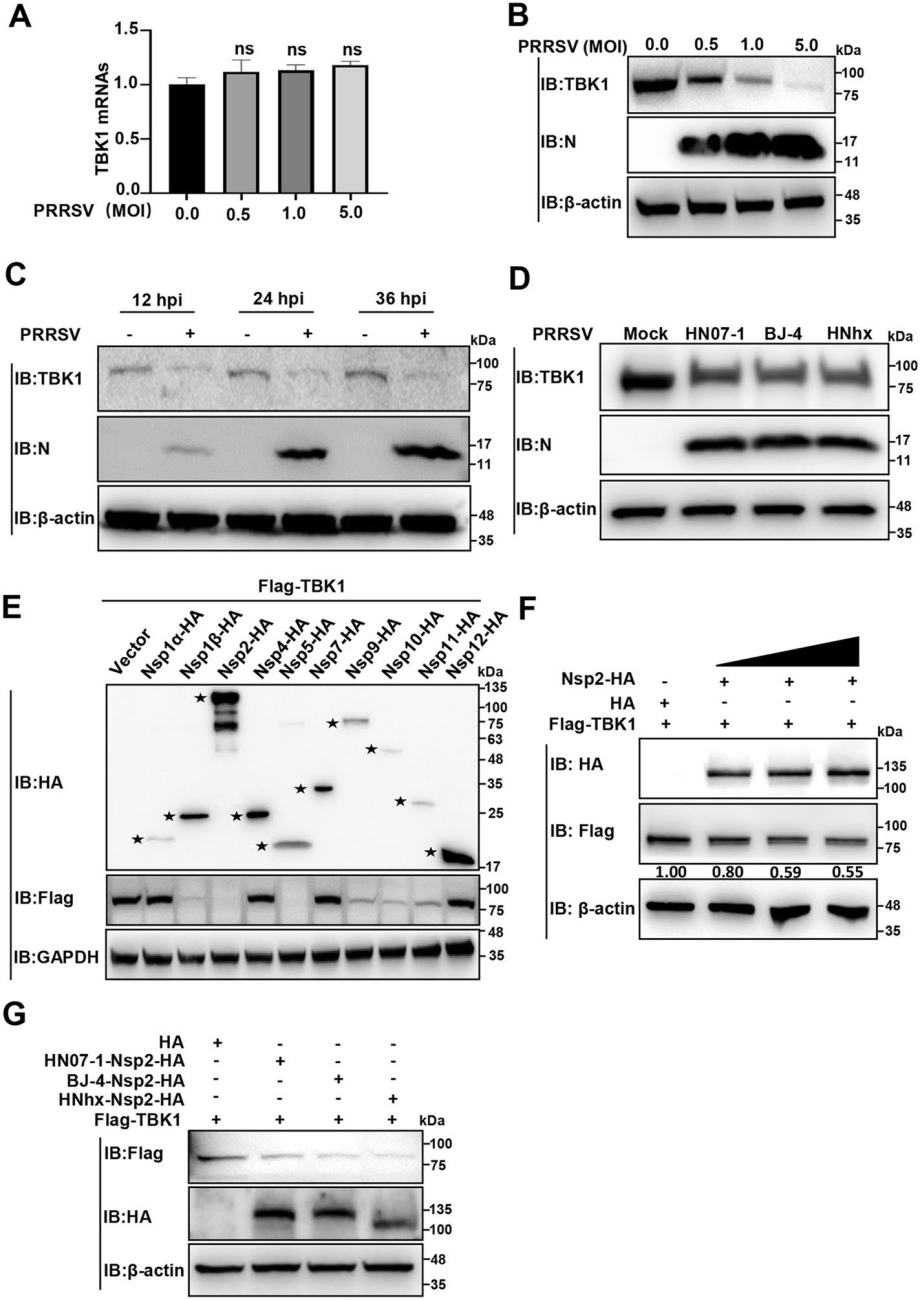
**PRRSV Nsp2 degrades TBK1**. **A**, **B** MARC-145 cells were infected with PRRSV strain HN07-1 at different MOIs (0.5, 1, and 5 MOI) or mock-infected and collected at 24 hpi. **A** The relative TBK1 mRNA abundance was analysed using RT-qPCR. Statistical analysis was carried out using the Student *t* test. ns, not significant (*P* > 0.05) **B** The TBK1 protein abundance was detected using IB. **C** MARC-145 cells were infected with PRRSV strain HN07-1 at an MOI of 1 or mock infected for indicated time periods (12, 24, or 36 h). The TBK1 protein level was detected by IB. **D** The mock-infected, or PRRSV strain HN07-1, BJ-4, or HNhx-infected (MOI = 1) MARC-145 cells were collected at 24 hpi. The TBK1 protein level was detected by IB. **E** HEK-293 T cells were transfected with the plasmids encoding each PRRSV strain HN07-1 Nsp-HA and Flag-TBK1 or HA-tagged empty vector, followed by IB analyses with the specific antibodies. Asterisks mark the target proteins. **F** The Nsp2-HA plasmid (0.5, 1, or 1.5 µg) was co-transfected with the Flag-TBK1 plasmid into HEK-293 T cells for 24 h. IB detected the TBK1 protein level. **G** HEK-293 T cells were transfected with the plasmids encoding Nsp2-HA of PRRSV strain HN07-1, BJ-4, or HNhx and Flag-TBK1 or HA-tagged empty vector, followed by IB analyses with the specific antibodies.

Firstly, we examined the RNA and protein levels of endogenous TBK1 in MARC-145 cells infected with the highly pathogenic (HP)-PRRSV strain HN07-1-infected using RT-qPCR and IB. As shown in Figures 1A and 1B, TBK1 RNA levels remained stable, while protein levels decreased significantly in a dose-dependent manner. We observed decreased TBK1 protein levels at 12, 24, and 36 h after HP-PRRSV infection (hpi, Figure 1C). Furthermore, we confirmed that the low pathogenic PRRSV strain BJ-4 and the NADC30-like PRRSV strain HNhx decreased TBK1 abundance (Figure 1D). All these results show that PRRSV infection degrades TBK1.

To identify which PRRSV Nsps degraded TBK1, we overexpressed each HA-tagged HP-PRRSV strain HN07-1 Nsp (Nsp-HA) and Flag-tagged-TBK1 (Flag-TBK1) in the HEK-293 T cells, and detected their expression and TBK1 protein levels. As shown in Figure 1E, Nsp1α-HA, Nsp1β-HA, Nsp2-HA, Nsp4-HA, Nsp5-HA, Nsp7-HA, and Nsp9-12-HA were successfully expressed, and among them, Nsp1β-HA, Nsp2-HA, Nsp5-HA, and Nsp9-11-HA degraded TBK1. As our laboratory has been investigating Nsp2 [39], we have proceeded with further research focusing on Nsp2-induced degradation of TBK1. We observed that the overexpression of HP-PRRSV Nsp2-HA led to a reduction in the abundance of exogenous TBK1 in a manner that depended on the dosage (Figure 1F). We verified that the Nsp2-HA of PRRSV strains BJ-4 and HNhx reduced TBK1 protein levels (Figure 1G). These data demonstrate that PRRSV Nsp2 degrades TBK1.

### PRRSV Nsp2 interacts with TBK1 and induces its degradation via lysosomal pathway

**Figure 2:**
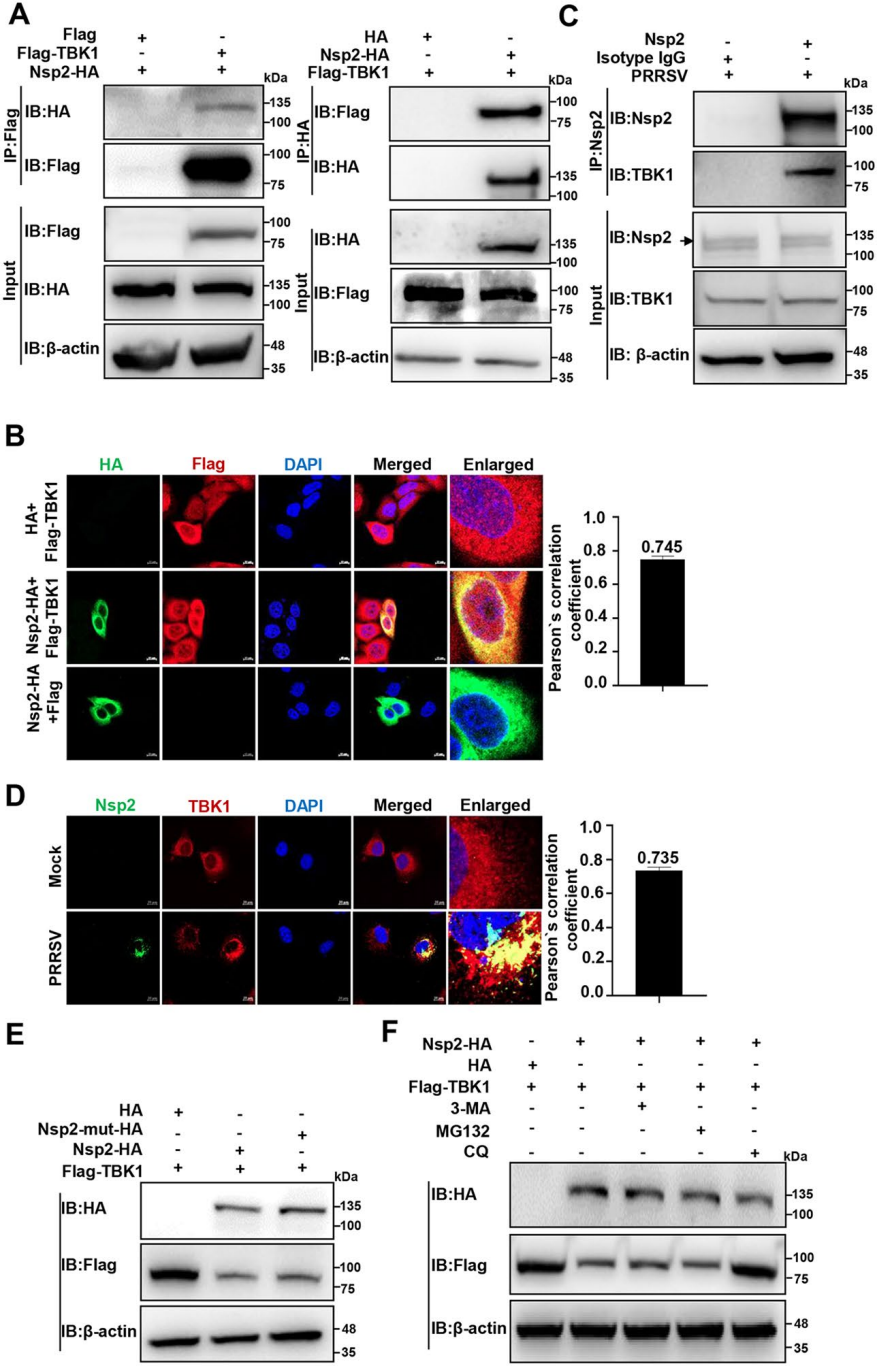
**PRRSV Nsp2 interacts with TBK1 and induces its degradation via the lysosomal pathway**. **A** HEK-293 T cells were transfected with the plasmids encoding Nsp2-HA and Flag-TBK1 or HA/Flag-tagged empty vector for 36 h, followed by co-IP with anti-HA/Flag magnetic beads and IB analyses with anti-HA/Flag antibodies.** B** HeLa cells were transfected with the plasmids encoding Nsp2-HA and Flag-TBK1 for 24 h. In parallel, HeLa cells were transfected with the plasmid encoding Nsp2-HA and Flag-tagged empty vector, or Flag-TBK1 and HA-tagged empty vector. Flag-TBK1 and Nsp2-HA were visualised with the specific primary and secondary antibodies. Cell nuclei were stained with DAPI. The fluorescent signals were observed with confocal microscopy (scale bars = 10 µm). The co-localisation was assessed by determination of the Pearson’s correlation coefficient using the JaCoP plugin in ImageJ software. **C** MARC-145 cells were infected with PRRSV at an MOI of 1 for 24 h. They were then analysed via endogenous IP using protein A/G magnetic beads pre-incubated with anti-Nsp2 pAbs, and IB with anti-Nsp2 and anti-TBK1 antibodies. **D** MARC-145 cells were infected with PRRSV at an MOI of 0.1 for 24 h. The fluorescent signals were observed with confocal microscopy (scale bars = 10 µm). The co-localisation was assessed by determining the Pearson’s correlation coefficient using the JaCoP plugin in ImageJ software. **E** HEK-293 T cells were co-transfected with the plasmids encoding Flag-TBK1 and Nsp2-HA or Nsp2-mutant-HA. At 24 h post-transfection, the cell lysates were collected to analyse the TBK1 protein level with IB.** F** HEK-293 T cells were transfected with the plasmids encoding Nsp2-HA and Flag-TBK1 for 6 h. 3-MA, CQ, MG132, or DMSO was added and the samples were collected after 24 h for IB analyses with anti-HA and anti-Flag antibodies.

We next examined whether PRRSV Nsp2 interacted with TBK1. We co-transfected the Nsp2-HA and Flag-TBK1 plasmids into HEK-293 T cells and performed IP using anti-HA/Flag magnetic beads. As shown in Figure 2A, we detected an interaction between exogenous Nsp2-HA and Flag-TBK1. Meanwhile, we overexpressed Nsp2-HA and Flag-TBK1 in HeLa cells and observed their co-localisation via confocal microscopy. Their co-localisation was further quantified using Pearson’s correlation coefficient, and the mean value was 0.745, indicating an interaction between these two proteins (Figure 2B). In addition, we monitored that endogenous Nsp2 interacted with TBK1 by IP and confocal microscopy in the PRRSV-infected MARC-145 cells (Figures 2C, D). These results substantiate that PRRSV Nsp2 specifically interacts with TBK1.

Considering their interaction, we initially assessed whether Nsp2 directly degrades TBK1. The PRRSV Nsp2 cysteine protease domain possesses both trans- and cis-cleavage activities, with highly conserved C55, C111, H124, C142, and C147 being responsible for the activities [40]. We mutated these residues to alanine (named Nsp2-mutant-HA) and detected that Nsp2-mutant-HA still degraded TBK1 (Figure 2E), indicating that its degradation was independent of Nsp2 cysteine protease activity. As the autolysosomal pathway and ubiquitin–proteasome system are two main host cellular degradation routes [41, 42], we investigated whether PRRSV Nsp2 degrades TBK1 through these two routes using the autophagy inhibitor 3-MA, lysosomal inhibitor CQ, and the proteasome inhibitor MG132 [43–45]. As shown in Figure 2F, only CQ treatment reversed TBK1 degradation, suggesting that Nsp2 induces TBK1 degradation in a lysosome-dependent manner.

### PRRSV Nsp2 interacts with HSPA8

**Figure 3:**
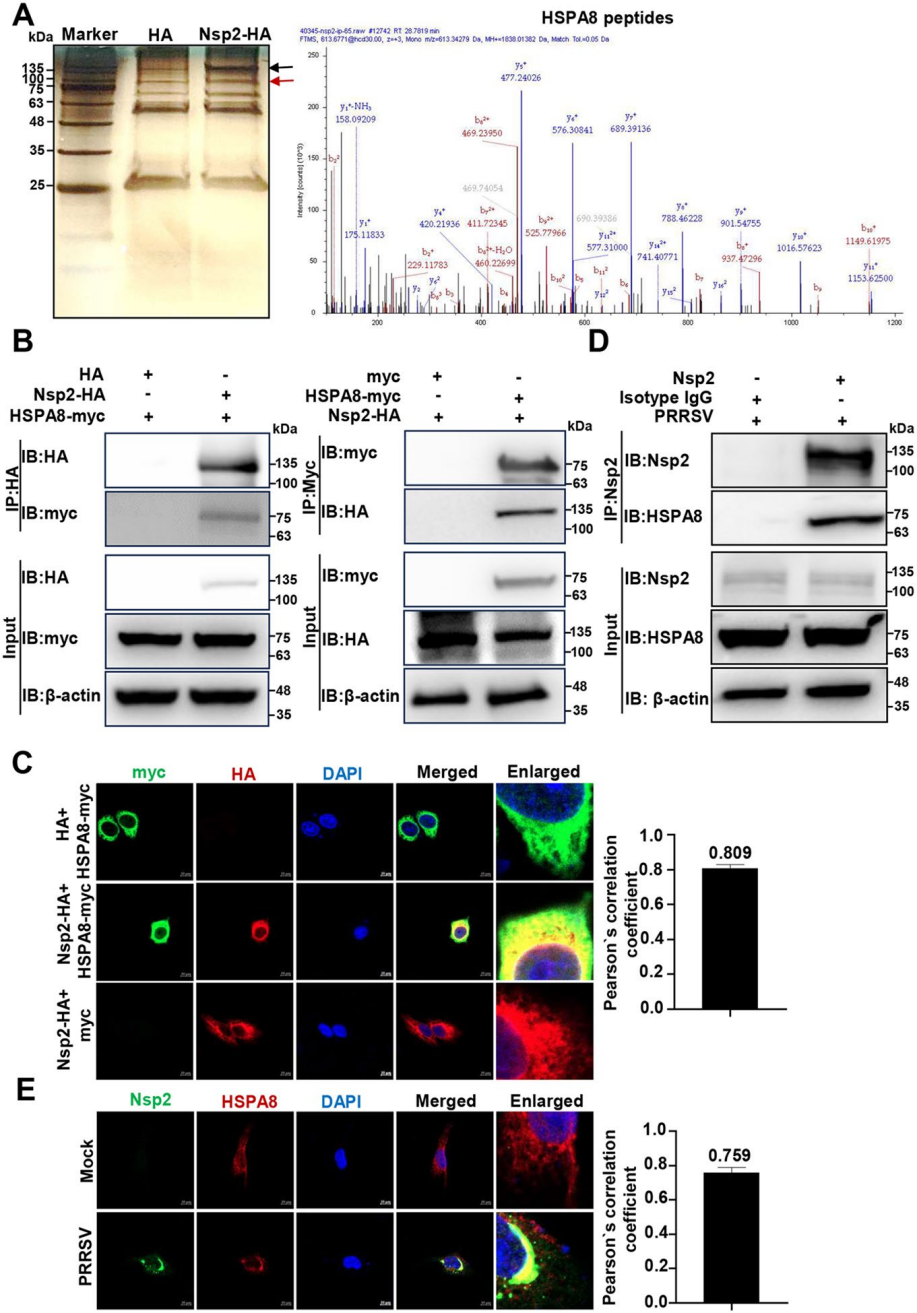
**PRRSV Nsp2 interacts with HSPA8**. **A** HEK-293 T cells were transfected with the plasmid expressing HA or Nsp2-HA. The proteins were immunoprecipitated in cell lysates using an anti-HA antibody, separated by 12% SDS-PAGE, and stained with silver. The red arrow indicates the significantly different immunoprecipitated protein band. The black arrow marks Nsp2-HA. The panel on the right shows the tandem MS analysis of HSPA8 peptides. **B** HEK-293 T cells were transfected with the plasmids encoding Nsp2-HA and HSPA8-myc or HA/myc-tagged empty vector for 36 h, followed by co-IP with anti-HA or anti-myc magnetic beads, and IB analyses with anti-HA and anti-myc antibodies. **C** HeLa cells were transfected with the plasmids encoding Nsp2-HA and HSPA8-myc for 24 h. In parallel, HeLa cells were transfected with the plasmid encoding Nsp2-HA and myc-tagged empty vector, or HSPA8-myc and HA-tagged empty vector. HSPA8-myc and Nsp2-HA were visualised with the specific primary and secondary antibodies. Cell nuclei were stained with DAPI. The fluorescent signals were observed with confocal microscopy (scale bars = 10 µm). The co-localisation was assessed by determination of the Pearson’s correlation coefficient using the JaCoP plugin in ImageJ software. **D** MARC-145 cells were infected with PRRSV at an MOI of 1 for 24 h. They were then analysed via endogenous IP using protein A/G magnetic beads pre-incubated with anti-Nsp2 pAbs, and IB with anti-Nsp2 and anti-HSPA8 antibodies. **E** MARC-145 cells were infected with PRRSV at an MOI of 1 for 24 h. The fluorescent signals were observed with confocal microscopy (scale bars = 10 µm). The co-localisation was assessed by determination of the Pearson’s correlation coefficient using the JaCoP plugin in ImageJ software.

To understand how Nsp2 breaks down TBK1 using lysosomes, we screened the host cellular proteins targeted by PRRSV Nsp2. We used IP along with LC–MS/MS. In cells overexpressing Nsp2-HA, we observed a distinct protein band marked by a red arrow (~71 kDa), identified as HSPA8 (Figure 3A).

To confirm that Nsp2 interacted with HSPA8, we co-transfected the Nsp2-HA and HSPA8-myc plasmids in HEK-293 T cells and detected an interaction between exogenous Nsp2 and HSPA8 (Figure 3B). Furthermore, we overexpressed Nsp2-HA and HSPA8-myc in HeLa cells and observed their co-localisation via confocal microscopy (Figure 3C). Moreover, we conducted IP and confocal microscopy in the PRRSV-infected cells and monitored endogenous Nsp2 interacting with HSPA8 (Figures 3D, E). These data indicate that PRRSV Nsp2 specifically interacts with HSPA8.

### PRRSV Nsp2 degrades TBK1 via chaperone-mediated autophagy (CMA)

**Figure 4:**
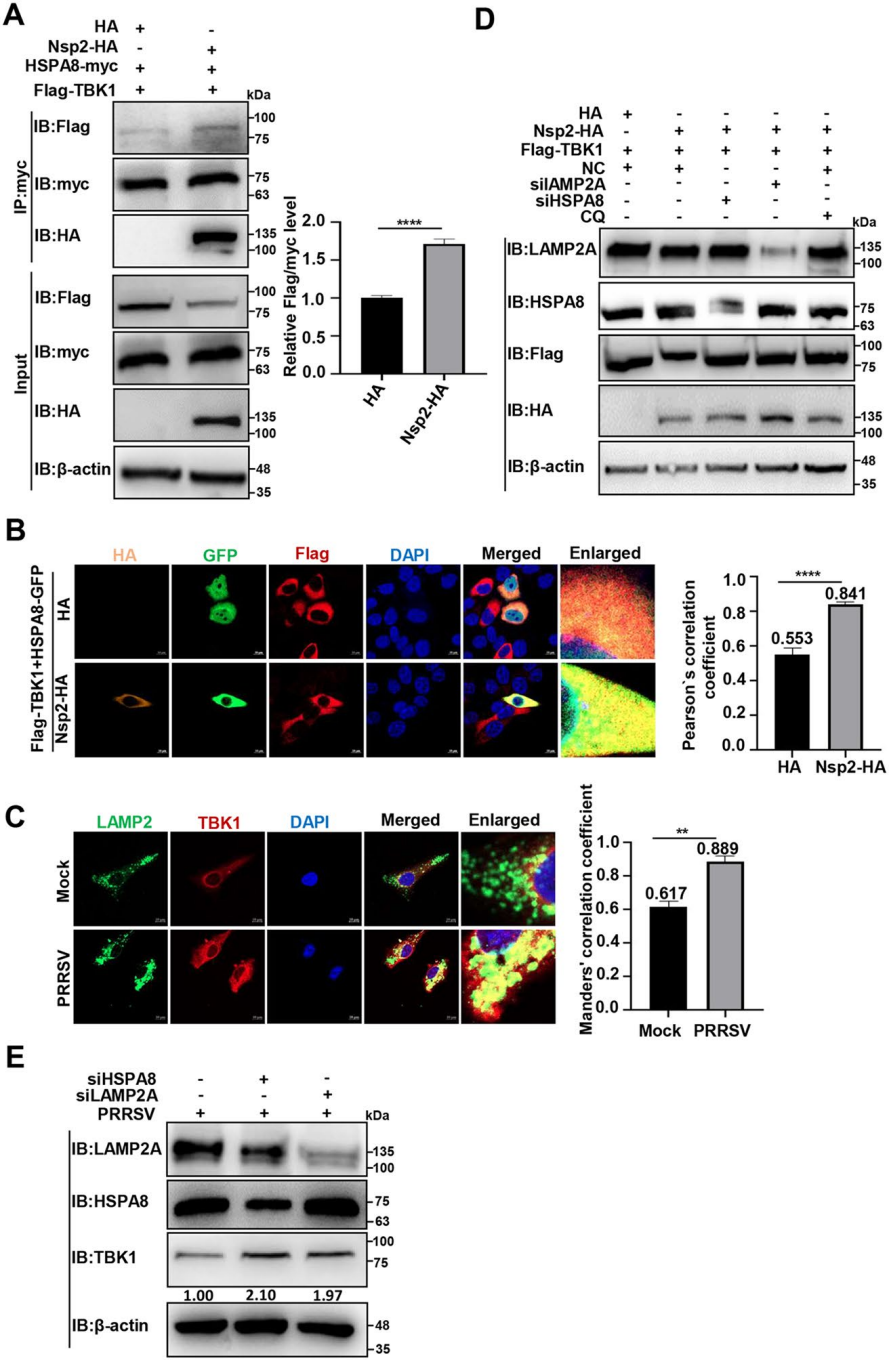
**PRRSV Nsp2 degrades TBK1 via CMA**. **A** HEK-293 T cells were transfected with the plasmids encoding Flag-TBK1, HSPA8-myc, and Nsp2-HA or HA-tagged empty vector for 36 h. Co-IP was performed with anti-myc magnetic beads and IB was conducted with the specific antibodies. **B** HeLa cells were transfected with the plasmids encoding Flag-TBK1, HSPA8-myc, and Nsp2-HA or HA-tagged empty vector for 36 h. The fluorescent signals were observed with confocal microscopy. The co-localisation was assessed by determination of the Pearson’s correlation coefficient (scale bars = 10 µm) using the JaCoP plugin in ImageJ software.** C** MARC-145 cells were infected with PRRSV at 0.1 MOI for 24 h. The fluorescent signals were observed with confocal microscopy. The co-localisation was assessed by determination of the Manders’ correlation coefficient (scale bars = 10 µm) using the JaCoP plugin in ImageJ software.** D** HEK-293 T cells were transfected with siHSPA8, siLAMP2A, or siNC, and the plasmids encoding Flag-TBK1 and Nsp2-HA. The samples were collected after 36 h for IB analyses with the specific antibodies. **E** MARC-145 cells were transfected with siHSPA8/LAMP2A or siNC to detect their effects during PRRSV infection, and IB was conducted with the specific antibodies. The data are presented as means ± SEM from three independent experiments. Statistical analysis was carried out using the Student* t* test. **, *P* < 0.01, ****, *P* < 0.0001.

HSPA8 recognises the Lys-Phe-Glu-Arg-Gln (KFERQ) motif on target proteins and forms HSPA8-substrate complexes; the complexes are subsequently translocated into lysosomes via LAMP2A for degradation, namely CMA [46]. It has been reported that HSPA8 interacts with TBK1 and degrades it via CMA [47]. Therefore, we hypothesised that Nsp2 enhances the interaction between HSPA8 and TBK1 to promote its degradation. We detected that Nsp2-HA overexpression strengthened the interaction of HSPA8-myc with Flag-TBK1 (Figures 4A, B). We also found that TBK1 localisation in lysosomes increased in PRRSV-infected MARC-145 cells (the Manders’ correlation coefficient increased from 0.617 to 0.889, Figure 4C). We then applied siHSPA8 or siLAMP2A and found that the knockdown of HSPA8 and LAMP2A reversed TBK1 degradation by Nsp2-HA (Figure 4D). Similarly, TBK1 degradation was antagonised during PRRSV infection in the *HSPA8* and *LAMP2A* knockdown cells (Figure 4E). Taken together, these data provide evidence that Nsp2 degrades TBK1 via CMA.

### PRRSV Nsp2 degrades TBK1 via CMA to suppress IRF3 activation and IFN production

**Figure 5:**
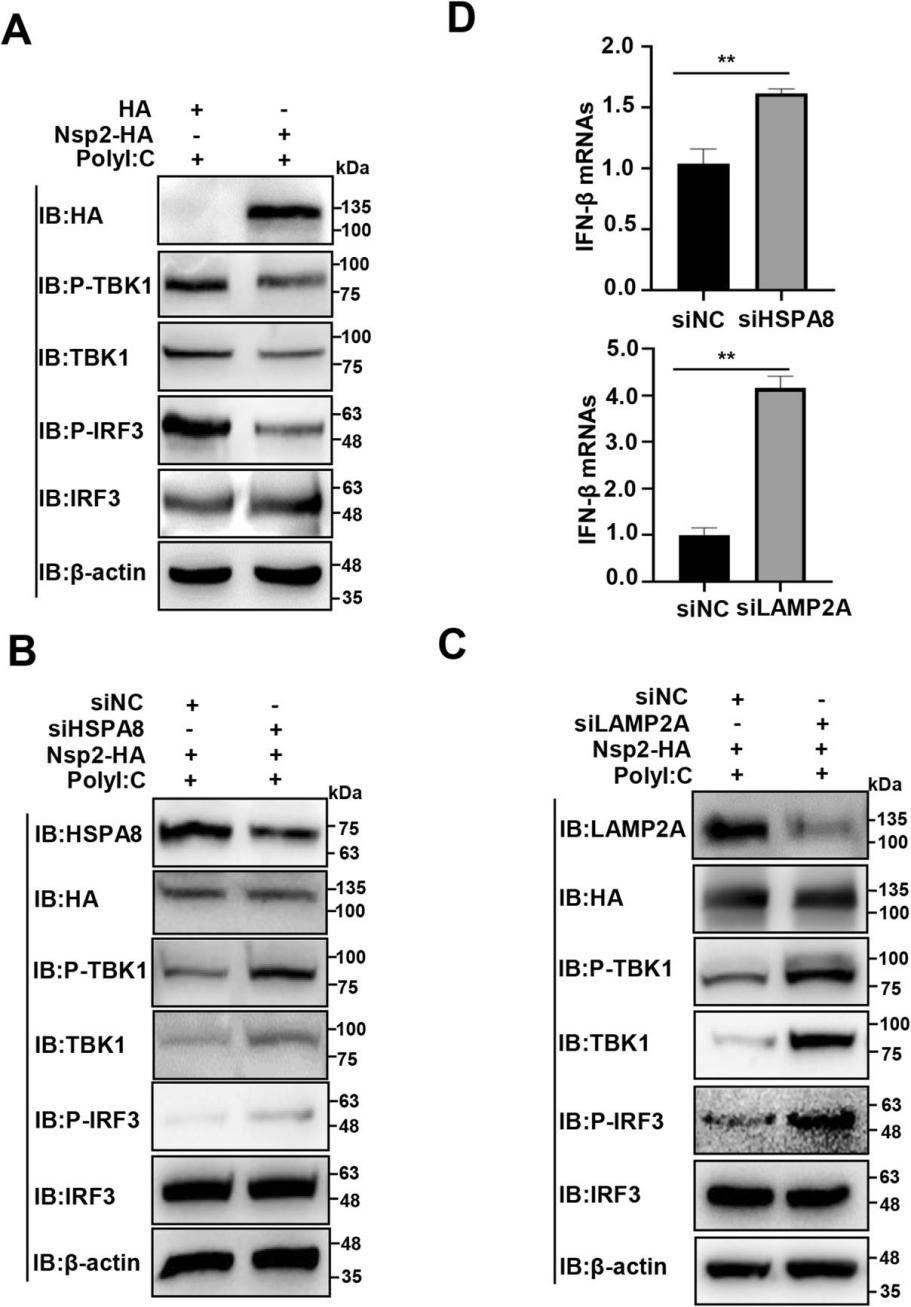
**PRRSV Nsp2 degrades TBK1 via CMA to suppress IRF3 activation and IFN production**. **A** HEK-293 T cells were transfected with the plasmid encoding Nsp2-HA or HA-tagged empty vector. The PolyI:C was added 12 h before the sample was collected. IB was conducted with the specific antibodies.** B**, **C** HEK-293 T cells were transfected with siHSPA8, siLAMP2A, or siNC, and the plasmid encoding Nsp2-HA. The PolyI:C was added 12 h before the sample was collected. IB was conducted with the specific antibodies.** D** HEK-293 T cells were transfected with siHSPA8, siLAMP2A, or siNC, and the plasmid encoding Nsp2-HA. The PolyI:C was added 12 h before the sample was collected. IFN-β RNA abundance was detected using RT-qPCR. The data are presented as means ± SEM from three independent experiments. Statistical analysis was carried out using the Student *t* test. **, *P* < 0.01.

TBK1 activates IRF3 signalling and initiates IFN production [48]. Consequently, we speculated that TBK1 degradation by Nsp2 via CMA hampered the p-TBK1 level, thereby hindering p-IRF3 levels and IFN production. As expected, overexpression of Nsp2-HA decreased the TBK1, p-TBK1, and p-IRF3 levels (Figure 5A). In contrast, the knockdown of HSPA8 and LAMP2A reversed the Nsp2-HA-induced degradation of TBK1 and increased the p-TBK1 and p-IRF3 levels (Figures 5B, C). Additionally, *HSPA8* and *LAMP2A* knockdown enhanced IFN-β production (Figure 5D). These data show that PRRSV Nsp2 degrades TBK1 through CMA to inhibit IRF3 activation and IFN production.

### PRRSV degrades TBK1 via CMA to suppress innate immunity and promote viral proliferation

**Figure 6:**
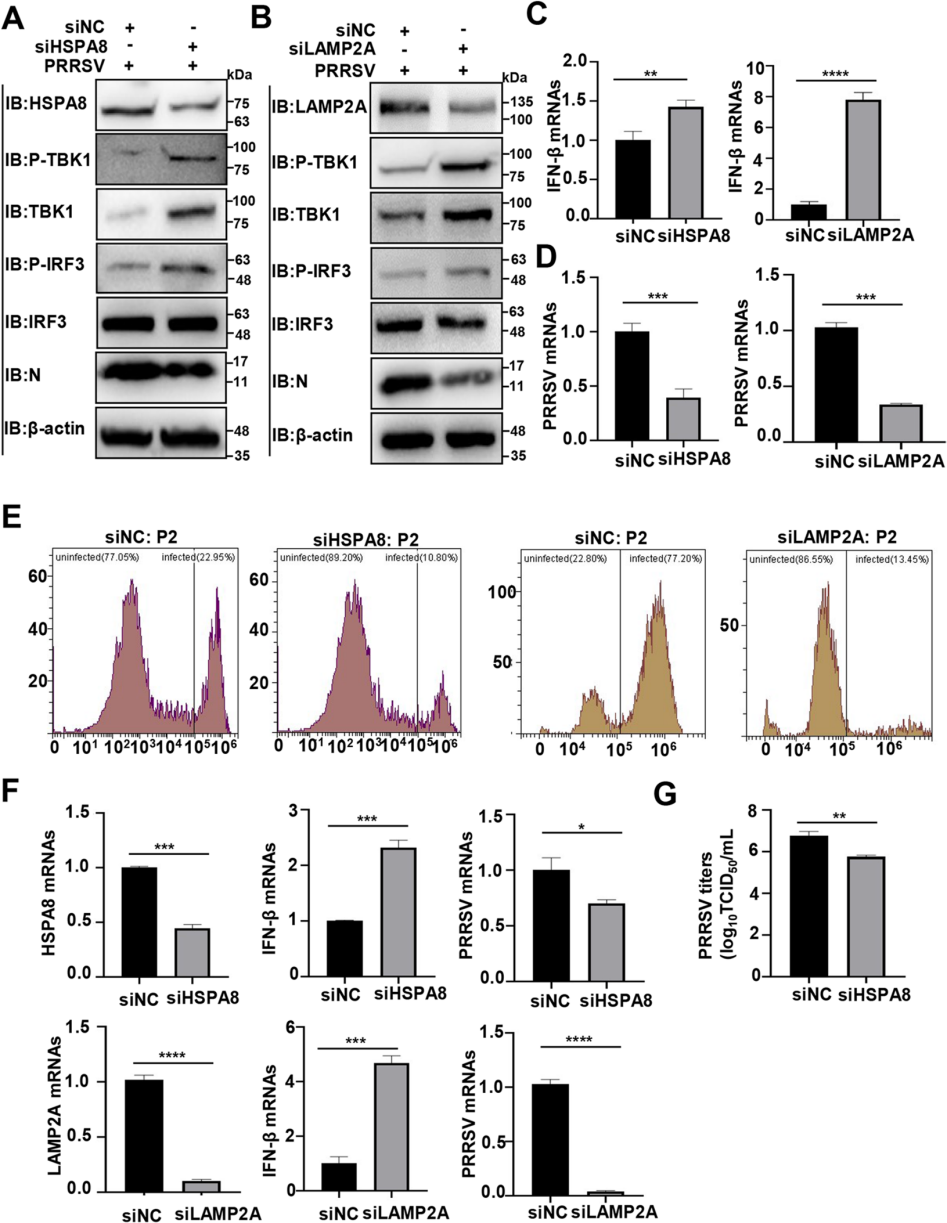
**PRRSV degrades TBK1 via CMA to suppress innate immunity and facilitate viral proliferation**. **A** MARC-145 cells were infected with PRRSV at an MOI of 1 for 2 h, then transfected with siHSPA8 or siNC for 48 h, and IB was conducted with the specific antibodies. **B** MARC-145 cells were transfected with siLAMP2A or siNC for 24 h. At 24 h post-transfection, the cells were infected with PRRSV at an MOI of 1 and IB was conducted with the specific antibodies. **C**–**E** MARC-145 cells were transfected with siHSPA8/siLAMP2A or siNC to detect their effects during PRRSV infection. **C** IFN-β RNA abundance was detected using RT-qPCR. **D** PRRSV RNA abundance was detected using RT-qPCR. **E** PRRSV infection was detected using FCM. **F** CRL-2843-CD163 cells were transfected with siHSPA8/siLAMP2A and the cell lysates were collected to detect their effects during PRRSV infection. IFN-β RNA and PRRSV RNA abundance were detected using RT-qPCR. **G** CRL-2843-CD163 cells were transfected with siHSPA8 or siNC. PRRSV titers were measured by assessing TCID_50_. The data are presented as means ± SEM from three independent experiments. Statistical analysis was carried out using the Student *t* test. *, *P* < 0.05; **, *P* < 0.01; ***, *P* < 0.0011; ****, *P* < 0.0001.

We further investigated the impact of PRRSV-induced TBK1 degradation via CMA on the host's innate immunity and viral proliferation. Figures 6A, B show that the knockdown of HSPA8/LAMP2A enhanced the TBK1, p-TBK1, and p-IRF3 levels during PRRSV infection. Meanwhile, *HSPA8*/*LAMP2A* knockdown increased IFN-β mRNA abundance while decreasing PRRSV RNA levels, as detected by RT-qPCR (Figures 6C, D). FCM results also showed that intracellular PRRSV infection was lowered (~50% reduction in the *HSPA8* knockdown cells and ~80% reduction in the *LAMP2A* knockdown ones, Figure 6E). As PAMs are primary in vivo target cells for PRRSV infection, we further performed the assays in a continuous PAM cell line CRL-2843-CD163, which stably expresses PRRSV indispensable receptor CD163 [49, 50]. Similarly, siHSPA8/siLAMP2A increased IFN-β RNA abundance and decreased PRRSV RNA levels (Figure 6F). The knockdown of HSPA8 also reduced PRRSV titres by at least tenfold (> 1 log_10_TCID_50_ mL^−1^, Figure 6G). These results show that PRRSV degrades TBK1 through CMA to suppress innate immunity and facilitate viral proliferation.

## Discussion

PRRSV employs various strategies to suppress innate immunity and establish persistent infection, posing a major obstacle to PRRS eradication [14]. Therefore, it is crucial to understand the interaction between PRRSV and the host’s innate immunity to elucidate PRRSV pathogenesis and improve prevention and control strategies. PRRSV Nsps antagonise the host’s innate immune responses [51]. However, there are limited studies on their antagonism of TBK1-mediated IFN production.

TBK1 is a kinase that mediates innate immune signalling. TBK1 phosphorylation leads to downstream IRF3 activation and IFN-I responses [52, 53], and is strictly regulated by several mechanisms [48, 54]. For example, the E3 ubiquitin ligase DTX4 promotes the polyubiquitination of TBK1 at Lys670 with K48 linkage, resulting in TBK1 degradation and the inhibition of immune responses [55].

In this study, we observed that different PRRSV strains degrade TBK1, and we further identified that PRRSV Nsp2 degrades TBK1 (Figure 1). Nsp2 carries out papain-like cysteine protease activities and cleaves viral polyproteins to produce Nsps as well as other target proteins for PRRSV replication [39, 40]. Interestingly, we found that PRRSV Nsp2 interacted with TBK1 but degraded TBK1 independent of its cysteine protease activity (Figure 2). Subsequently, we showed that PRRSV Nsp2 degrades TBK1 via CMA, which reveals a new mechanism for protein degradation by Nsp2.

CMA is a form of selective autophagy, where proteins containing the KFERQ motif are recognised by HSPA8 and then translocated into lysosomes via LAMP2A for degradation [46]. It has been shown to participate in TBK1 degradation [47]. We confirmed that PRRSV Nsp2 interacts with HSPA8 and enhances its interaction with TBK1 to degrade TBK1 via CMA (Figures 3 and 4).

Finally, PRRSV Nsp2-induced TBK1 degradation via CMA decreased TBK1 phosphorylation levels and inhibited downstream IRF3 activation. This suppressed IFN-I production and promoted viral proliferation (Figures 5 and 6). PRRSV Nsp2 is well-known for its involvement in immunosuppression [26, 56–58]. In detail, Nsp2 impedes the transport of stimulator of IFN genes from the endoplasmic reticulum to the Golgi apparatus and blocks IFN signalling [56]. In addition, Nsp2 inhibits the signalling of melanoma differentiation-associated gene 5, which impairs immune responses [57]. Nsp2 also antagonises NF-κB-mediated immune responses by deubiquitinating K48-linked I-kappa-B-alpha [58]. Moreover, Nsp2 prevents the phosphorylation and nuclear translocation of IRF3, thereby reducing the expression of IFN-I [26]. Our study reveals a different mechanism through which Nsp2 suppresses the host’s innate immunity.

**Figure 7:**
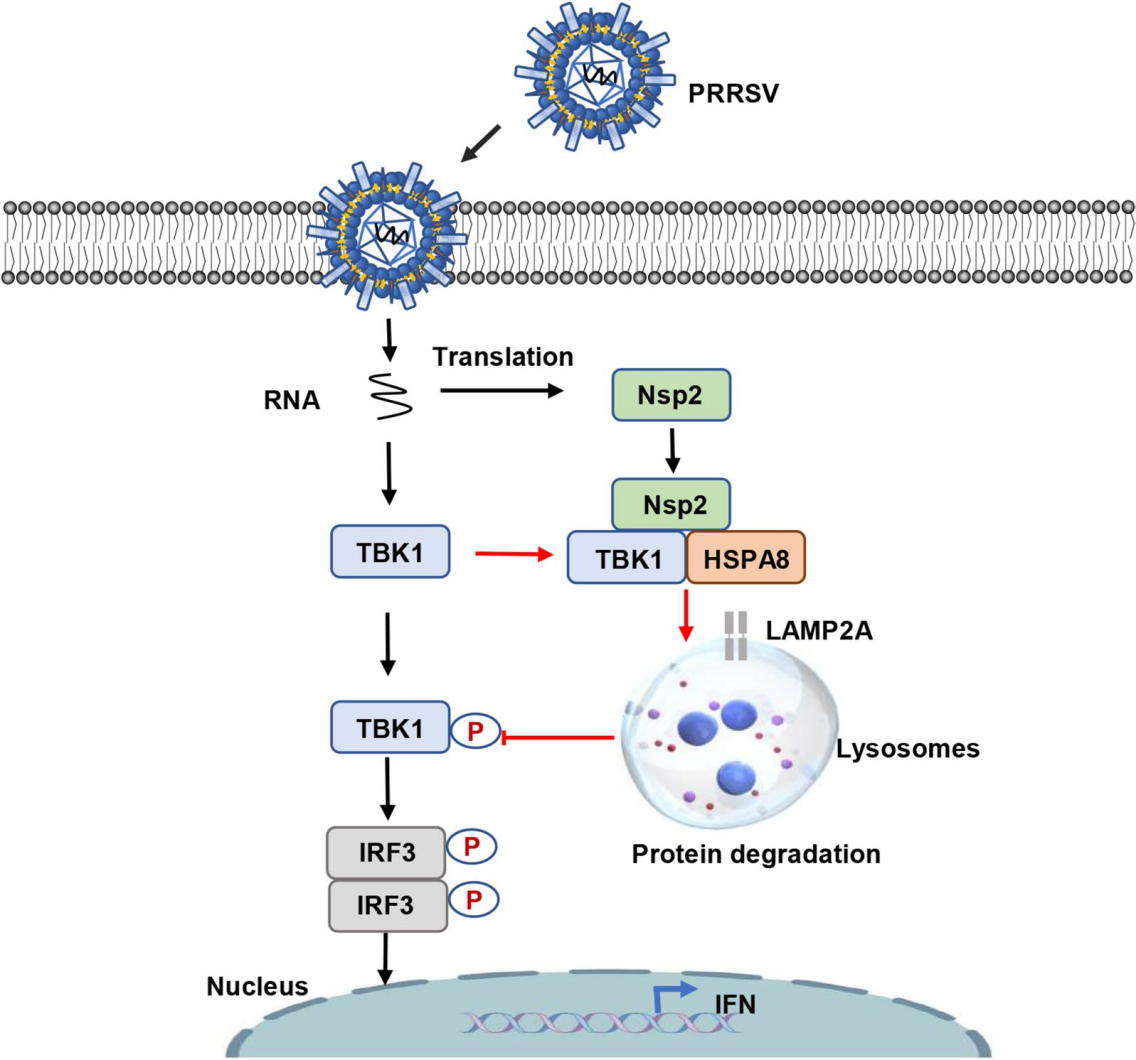
**Schematic model depicting that PRRSV inhibits IFN-I production by degrading TBK1 via CMA, thereby promoting viral proliferation**. Mechanistically, PRRSV Nsp2 enhances the interaction between HSPA8 and TBK1, leading to LAMP2A-mediated translocation of TBK1 into lysosomes for degradation, which impedes downstream IRF3 signalling and IFN-I production.

Based on these results, we propose a model to describe how PRRSV degrades TBK1 via CMA to suppress host innate immunity and facilitate viral proliferation (Figure 7). Our results contribute to a comprehensive understanding of PRRSV immunosuppression and provide promising targets for the prevention and control of PRRS.

## Data Availability

The data generated during this study are available from the corresponding authors upon reasonable request.

## Abbreviations

PRRSV: porcine reproductive and respiratory syndrome virus
IFN-I: type I interferon
TBK1: TANK-binding kinase 1
CMA: chaperon-mediated autophagy
Nsp2: nonstructural protein 2
HSPA8: heat shock protein member 8
LAMP2A: lysosomal-associated membrane protein 2A
IRF3: IFN regulatory factor 3
NF-κB: nuclear factor kappa-light-chain-enhancer of activated B cells
PRRS: porcine reproductive and respiratory syndrome
PAMs: porcine alveolar macrophages
HEK-293 T: human embryonic kidney 293 T
FBS: fetal bovine serum
mAb: monoclonal antibody
p-TBK1: phospho-TBK1
p-IRF3: phospho-IRF3
HA: hemagglutinin
pAbs: polyclonal antibodies
GAPDH: glyceraldehyde-3-phosphate dehydrogenase
HRP: horseradish peroxidase
N: nucleocapsid
IB: immunoblotting
RIPA: radioimmunoprecipitation assay
WB/IP: Western blot/immunoprecipitation
DAPI: 4′,6-Diamidino-2-phenylindole
ECL: enhanced chemiluminescence
RT-qPCR: quantitative real-time PCR
SDS-PAGE: sodium dodecyl sulfate–polyacrylamide gel electrophoresis
RT: room temperature
MS: mass spectrometry
LC–MS/MS: liquid chromatography and tandem MS
siRNA: small interference RNA
MOI: multiplicity of infection
siNC: SiRNA-negative control
FCM: flow cytometry
TCID_50_: 50% Tissue culture infected dose
SEM: standard error of the mean
hpi: hours post-infection
